# Supplementary material for: Effectiveness of antimalarial drug combinations in treating concomitant urogenital schistosomiasis in malaria patients in Lambaréné, Gabon: A non-randomised event-monitoring study
Source: PLoS Negl Trop Dis. 2022 Oct 31;16(10):e0010899. doi: 10.1371/journal.pntd.0010899 (PMC9648843; doi:10.1371/journal.pntd.0010899)
Supplement: S1 Study Protocol — (PDF) [file pntd.0010899.s003.pdf]

Centre de Recherches Médicales de Lambaréné

www.cermel.org
BP 242 · Lambaréné
BP 1437 · Libreville
Gabon
Tel: +241 07 98 91 91
admin@cermel.org

Study Protocol

Evaluation of the effect of artemisinin-based combination therapies on urinary *Schistosoma haematobium* when administered for the treatment of concomitant malaria

Protocol version 1.1 (03-03-2018)

Principal Investigator : Rella Zoleko Manego, MD

Co-Investigators:

Michael Ramharter, MD

Ghyslain Mombo-Ngoma, MD PhD

Luzia Valentzky, MD

Lia Betty Dimessa, MD

Malik Azeez Akinosho, MD

Daniel Stelzl, MD

Akim Ayola Adegnika, MD PhD

Maxime Selidji Agnandji, MD PhD

Centre de Recherches Médicales de Lambaréné (CERMEL)

B.P. 242, Lambaréné

Gabon

www.cermel.org
BP 242 · Lambaréné
BP 1437 · Libreville
Gabon
Tel: +241 07 98 91 91
admin@cermel.org

1 Background

Schistosomiasis is among the most prevalent water borne diseases, and it is globally the second most prevalent parasitic infection after malaria in the tropics and subtropics areas [1 2]. According to the World Health Organization (WHO), it is a disease that affects 230 million people worldwide, of which 90% of cases live in Africa [3]. The sanitary system and the hygiene of the population play an essential role in the development of the disease. The 6th international congress of tropical medicine on health and urbanization in Africa held in Senegal in 2001 explained this expansion by the development of agglomerations characterized by a disordered urbanization. Studies in Gabon have shown that the prevalence of schistosomiasis varies by region. Recently, Ateba et al confirmed during the work conducted in 2013 around Lambaréné that children represented the most infected population as well as the one with the most intense infection. In fact, 43% of the children were infected with Schistosoma heamatobium [4].

Artemisinin has been used successfully in the treatment of malaria infections, usually in combination with another antimalarial to prevent the development of parasite resistance. In 2007, a study showed the efficacy of artemisinin-based combination therapies (ACTs) in the management of schistosomiasis when administered in the treatment of uncomplicated malaria [5]. In 2008 mefloquine, used as intermittent preventive therapy against malaria in pregnancy (IPTp), showed high egg reduction rates in women with a concomitant *S. haematobium* infection [6] In 2004, a study showed that artesunate would be more cost-effective than PZQ in terms of cost effectiveness [7]. ACTs could be combined with PZQ thus targeting different stages of parasite development to improve treatment outcomes. The evaluation of the efficacy of artemisinin derivatives in the treatment of schistosomes is therefore of great importance. This is easily understood by the fact that these products are commonly used against malaria, and secondly because malaria and schistosomiasis are two conditions that are both endemic in the same regions with the possibility of co-infection. Praziquantel (PZQ) is the drug of choice for treating all forms of schistosomiasis. However this drug is not effective against immature stages and young parasites. In addition, the problem of parasite resistance and the rapidity of reinfection require the development of new effective therapies

www.cermel.org
BP 242 · Lambaréné
BP 1437 · Libreville
Gabon
Tel: +241 07 98 91 91
admin@cermel.org

2 Objectives of the study

The aim of this study is to determine whether artemisinin-based combination therapies (ACTs) given for the treatment of uncomplicated malaria can cure concomitant infection with *Schistosoma haematobium*.

2.1 Primary objective

The primary objective is to evaluate egg reduction conferred by ACTs as treatment of infection with *Schistosoma haematobium* while administered for the treatment of concomitant malaria.

2.2 Secondary objectives

Cure rate of ACTs for *S. haematobium* infection

Comparison of ACTs versus all non-artemisinin therapies currently under development and evaluated at Cermel.

3 Time and place of Study

The study will be conducted at the Centre de Recherches Médicales de Lambaréné (CERMEL). The study will be done between Mars 2018 and December 2019

4 Study population

Context

All study participants for this study will be recruited amongst study subjects enrolled in the ongoing malaria clinical trials and malaria patients routinely treated at the CERMEL. Briefly, there are several clinical trials on antimalarial drugs conducted at CERMEL and the currently investigated drugs are PYRAMAX (artesunate-pironaridine) in a phase IIIb/IVstudy , KAF156 and lumefantrine solid dispersion formulation, artefenomel (OZ439) and ferroquine,

www.cermel.org
BP 242 · Lambaréné
BP 1437 · Libreville
Gabon
Tel: +241 07 98 91 91
admin@cermel.org

Ferroquine alone and KAE609 alone in phase II studies. Rescue treatments are either artemether-lumefantrine or artesunate-amodiaquine. The subjects enrolled within these clinical trials are of all ages and both sexes. The study is longitudinal in design with two groups and based in the Tsamba-Magotsi department, Ngounié province and in town of Lambaréné Moyen-Ogoue province.

Study design

The study is designed as an open label controlled non-randomized trial to assess the efficacy and safety of artemisinin derivatives versus non-artemisinin drugs on *S. haematobium* infection while administered for the treatment of malaria.
The laboratory assessor will be blinded regarding the treatment received by the subject.

Description of study population

Individuals of all ages diagnosed with malaria will be invited during a consent process to participate in the current study by screening of urine for schistosomiasis and only those positive for *S. haematobium* will be enrolled in the study.

Inclusion criteria

Persons of all ages and both sexes and residing in the study area are eligible for study participation. The following criteria are also required:

- Malaria infection diagnosed by RDT or thick blood smear
- Urinary schistosomiasis diagnosed by presence of *Schistosoma haematobium* eggs in the urine before malaria treatment
- Written informed consent given

Exclusion criteria

- Patients treated with Praziquantel during the 6 previous weeks
- Known intolerance or allergy to any study drug
- Pregnancy

www.cermel.org
BP 242 · Lambaréné
BP 1437 · Libreville
Gabon
Tel: +241 07 98 91 91
admin@cermel.org

Sample size

The choice of an adequate sample size for this study is mainly based on practical considerations which state that a sample of 50 patients positive for schistosoma by the investigation is sufficient to evaluate egg reduction rates of the investigated drug [8]. To calculate the sample size we estimated the egg reduction rate at 80% with artemisinin derivates and 40% with new combination drug. With a power of 90% and an alpha error of 5% we needed 30 subjects in each group. An additional 5 subjects were added to each group to correct any lost to follow-up and non-compliance by patients. So a total of 70 subjects are required.

Study treatment

ACTs and non-artemisinin drugs and drug combinations will be administered following the primary indication for treatment, which is for the treatment of uncomplicated malaria Patients will not be randomized in this observational study.

5 Endpoints

5.1 Primary endpoint

Egg reduction rate at 6 weeks after initiation of antimalarial treatment

5.2 Secondary endpoint

Cure rate at days 28 and 42

Incidence of hematuria before and after treatment (macroscopic and microscopic)

Comparative efficacy ACTs versus non-ACT

Safety

Definition of cure rate and egg reduction

Cure rate (CR): Proportion of person with no *Schistosoma haematobium* egg at the post treatment follow-up. CR=100%×(1−number of subjects excreting eggs at follow-up/number of subjects excreting eggs at baseline)

www.cermel.org
BP 242 · Lambaréné
BP 1437 · Libreville
Gabon
Tel: +241 07 98 91 91
admin@cermel.org

Egg reduction rate (ERR): Proportion of person with the reduction in the number of eggs excreted. ERR=100%×(1−arithmetic mean of group at follow-up/arithmetic mean of group at baseline).

Absence of egg is definite as no egg count on two consecutive urine sampling.

6 Study procedure

Study related procedures

All individuals of all ages and both sexes having given an informed consent and are with the presence of eggs of *S. haematobium* in urine at screening will be eligible for the study. The screening will consist of 10 mL of urine freshly collected passed through a filter and diagnosed using a microscope. Basic demographic data will be recorded as well as the history of hematuria. During the follow up visits urine samples will be also collected on day 28 and day 42 (6 weeks posttreatment). The posttreatment assessments will be done on urine samples collective on at least 2 consecutive days. All subjects found with schistosomiasis will be treated with single dose of praziquantel (40 mg / kg) as recommended by the World Health Organization at the end of follow up. The follow up visit schedule will be the same in both groups.

Urine sampling

For each urine sampling, a macroscopic examination will be performed following by dipstick for screening of the presence of erythrocytes and leukocytes, then an urinary filtration and microscopy examination for the detection of eggs will be done.

7 Ethics

Informed consent

All volunteers will sign and date the informed consent form before any study specific procedures are performed.

www.cermel.org
BP 242 · Lambaréné
BP 1437 · Libreville
Gabon
Tel: +241 07 98 91 91
admin@cermel.org

Ethical review

This trial will be submitted for ethical review to the Institutional Review Board (Comité d'Ethique institutionel, CEI) of the Centre de Recherches Médicales de Lambaréné (CERMEL).

Risks and side effects

Benefits include diagnostic and provision of treatment for those subject found with the Schistosoma infection at the end of follow up visit. Potential risks include the fact that standard treatment is deferred until 6 weeks post diagnosis. On the background of treatment with investigational drugs for malaria it is justified to defer anti-schistosomal treatment to minimize risks for potential drug-drug interactions with regards to pharmacokinetics but also with regards to tolerability and safety.

Data protection

Each participant will receive a participant number (pseudonymized). The data will be entered in a database pseudonymously.

Patient compensation

No volunteer compensation will be provided.

www.cermel.org
BP 242 · Lambaréné
BP 1437 · Libreville
Gabon
Tel: +241 07 98 91 91
admin@cermel.org

References

1. OMS. 2011.
2. Engels, D., et al., The global epidemiological situation of schistosomiasis and new approaches to control and research. Acta Trop, 2002. 82(2): p. 139-46.
3. OMS, Schistosomiase, Aide-mémoire N°115. 2015, OMS: Geneve.
4. Ateba-Ngoa, U., et al., Assessment of the effect of Schistosoma heamatobium co infection on malaria parasites and immune responses in rural populations in Gabon: Study protocol. 2012.
5. Inyang-Etoh PC, Ejezie GC, Useh MF, Inyang-Etoh EC. Efficacy of artesunate in the treatment of urinary schistosomiasis in an endemic community in Nigeria. Ann Trop Med Par 2004; 98(5): 491-499
6. Basra A, Mombo-Ngoma G, Capan Melser M, Akerey Diop D, Würbel H, Mackanga JR, Fürstenau M, Manego Zoleko R, Adegnika AA, Gonzalez R, Menendez C, Kremsner PG, Ramharter M. Efficacy of Mefloquine Intermittent Preventive Treatment in Pregnancy Against Schistosoma haematobium Infection in Gabon: A Nested Randomized Controlled Assessor-Blinded Clinical Trial. Clin Infect Dis. 2013 Mar;56(6):e68-75.
7. Inyang-Etoh PC, Ejezie GC, Useh MF, Inyang-Etoh EC. Efficacy of artesunate in the treatment of urinary schistosomiasis in an endemic community in Nigeria. Ann Trop Med Par 2004; 98(5): 491-499

8. WHO 2013: Assessing the efficacy of anthelminthic drugs against schistosomiasis and soil-transmitted helminthiases.
