# Supplementary material for: Effectiveness of antimalarial drug combinations in treating concomitant urogenital schistosomiasis in malaria patients in Lambaréné, Gabon: A non-randomised event-monitoring study
Source: PLoS Negl Trop Dis. 2022 Oct 31;16(10):e0010899. doi: 10.1371/journal.pntd.0010899 (PMC9648843; doi:10.1371/journal.pntd.0010899)
Supplement: Attachment — Submitted filename: Reviewer_report.docx [file pntd.0010899.s004.docx]

**Effectiveness of antimalarial drug combinations in treating concomitant urogenital schistosomiasis in malaria patients in Lambaréné, Gabon: A non-randomised event monitoring study**

Thank you for allowing me to review the above titled manuscript. Authors assessed the cure rates and egg reduction rates of different artemisinin combination therapies against Schistosoma haematobium. This was an observational study which was mainly a secondary analysis from the primary objective of assessing the efficacy of at antimalarial in Gabon. In general, the manuscript is well written and the methodology description are very clear. Although the manuscript does not present new knowledge on the effects of artemisinin-based antimalaria on Schistosomes species, it does add more evidence on future use of these drugs and the advantage they have in individuals who are co-infected with malaria and schistosomiasis. I have minor observations:

- Methods: Follow the PLOS NTD Format
- Sample size: descriptive study, does Power make any sense?
- Urine sample collection: Did you allow patient to exercise to allow more eggs to be collected in the urinary bladder before sample collection?
- Did you note the time of sample collection, understanding the circadian circle of eggs release?
- Assessment of ERR and CR- The WHO reference recommends 14 days to 21 days post-treatment for evaluation of ERR and CR. Why did the author assess the two variables at 4- and 6-weeks post-treatment?
- Results? What determined allocation of participants in different antimalaria treatment?
- Did the author obtain ethical approval for this observational study?
