# Supplementary material for: Effectiveness of antimalarial drug combinations in treating concomitant urogenital schistosomiasis in malaria patients in Lambaréné, Gabon: A non-randomised event-monitoring study
Source: PLoS Negl Trop Dis. 2022 Oct 31;16(10):e0010899. doi: 10.1371/journal.pntd.0010899 (PMC9648843; doi:10.1371/journal.pntd.0010899)
Supplement: Attachment — Submitted filename: Point-by-point_responses_reviewers_13June2022.docx [file pntd.0010899.s005.docx]

| Bernhard Nocht Institute for Tropical Medicine  P.O. Box 50 06 40, D-22706 Hamburg, Germany |  | **Prof. Ghyslain Mombo-Ngoma, MD, MSc, PhD** |
| --- | --- | --- |
|  |  | Head of Implementation of Drugs Therapies  Department of Implementation Research & University Medical Centre Hamburg-Eppendorf |
|  |  |  |
|  |  | Bernhard-Nocht-Str. 74  20359 Hamburg, Germany |
|  | Tel | 040 42818-589 |
|  |  |  |
|  | E-Mail | ghyslain.mombo-ngoma@bnitm.de |
|  |  |  |
|  |  | Hamburg, 13 June 2022 |

__

Dear Editor,

We are grateful for the opportunity to resubmit a revised version of our manuscript. We are grateful for the reviewers’ comments which we have taken into consideration and which helped us to further improve our manuscript. Please find the point-to-point response below and the modified manuscript attached.

Kind regards,

Ghyslain MOMBO-NGOMA

Point-by-point Responses to Reviewer's Questions

>**Methods**

**Reviewer #1**: This is clear

**Reviewer #2**: Overall, the results presented are interesting. However, the data concerning artefenomel-ferroquine and artesunate-amodiaquine are not sufficiently relevant. They concern a small number of patients: older in the ASAQ group and too young in the OZFQ group compared to the AL and AP groups. I would suggest removing them.

**Response: Thank you for this comment. We agree with that and data of ASAQ and OZFQ groups were removed from the table.**

**Changes:**

**Table 1:** Baseline characteristics of study participants treated with artesunate-pyronaridine and artemether-lumefantrine and included in the analysis, N=39

**Table 3**

**Reviewer**: In terms of methodology, The diagnostic aspects, in particular the method of determining the number of eggs excreted, must be clearly presented. It is important to specify the method for determining the parasite load. Were all the eggs counted or a formula used? The readers of the article should find all this information in the methodology, this will allow a reproductibility of the used methodology. How was determined the egg viability at week 4 and week 6?

**Response: Thank you for this comment. The urine filtration method reported by Peters et al (1976) for counting of *Schistosoma haematobium* eggs in urine was used. This method is highly sensitive and reproducible, staining is not necessary, and the small size of the filters allows counts to be made at low power with great rapidity. Briefly, 10 ml of urine are injected through a PT-013 chamber containing a Nuclepore filter, 13 mm in diameter with a pore size of 8 µm. The syringe is removed and filled with air, which is injected through the filter chamber. The chamber is opened, and the filter removed and placed down on a glass slide. The counting is done using x40 magnification scanning completely the filters in 4 passes and the egg count per volume of urine used is recorded.**

**Reference:** Peters PA, Warren KS, Mahmoud AA. Rapid, accurate quantification of schistosome eggs via nuclepore filters. J Parasitol. 1976 Feb;62(1):154-5. PMID: 1255368.

**Egg viability was not determined at week 4 and week 6 and any potential bias induced by this limitation is assumed to be equally distributed between the treatment groups. This limitation was added in the discussion section.**

**Change:**

The following changes have been made to the methods section:

Line 173: The counting of eggs is done using x40 magnification scanning completely the filter in four passes and the egg count per volume of urine is recorded.

**Reviewer**: Were there patients not excluded for carriage of non-hematobium species or of hybrid Schistosoma sp whose carriage frequency in Gabon is not negligible? Although this is an observational study, patient selection criteria based on characteristics, such as the parasite load, should have been established because the difference in the median eggs count at baseline is significant between the groups (19 times higher in the AL group compared to the AP group). This has an impact on parasite

clearance at week 4 and week 6. Similarly, the molecules studied differ in the combinations used. Authors should discuss this more explicitly. How were patients who did not participate in a clinical trial selected?, Were there any participants with macroscopic hematuria? if yes, what

was the evolution of this hematuria after 6 weeks? The subdivision of the groups for the endpoint analysis at week 4 and week 6 needs to be clarified

**Response:**

**According to the review from Dejon-Agobé et al. (2022) S. haematobium is the main Schistosoma** [**species endemic**](https://www.sciencedirect.com/topics/immunology-and-microbiology/endemic-species) **in the study region, with the presence of only some cases of intestinal Schistosoma species. Whilst the differentiation between S. mansoni eggs from S. haematobium or S. guineensis eggs can be easily done morphologically by microscope examination, S. intercalatum reported from Lambaréné should be speciated at molecular level to be reliably differentiated from S. haematobium, since eggs from both species are morphologically similar (****[Almeda et al., 1996](https://www.sciencedirect.com/science/article/pii/S0001706X2200016X?via%3Dihub#bib0004)).**

**In our study, no *S. intercalatum* was diagnosed as this is mostly seen in stool samples. No molecular speciation was performed on the ground that it is unlikely to find *S. intercalatum* in urine. In addition, prevalence of S. intercalatum is considered lower than 1% and any potential bias by inclusion of S. intercalatum carriers is assumed to be evenly distributed all treatment groups. Thus, no patient was excluded for carriage of non-*haematobium* species.**

**We agree with the reviewer that patient median egg count at baseline is different between the groups and may have an impact on egg clearance at 4- and 6-weeks. This is a major limitation of this observational study which we acknowledge openly in the Discussion section. However, we did an analysis to check if schistosoma infection intensity may impact egg clearance at 4- and 6-weeks following antimalarial drug administration and the result did not support that hypothesis.**

**Inclusion criteria were not different between participants who did not participate in the clinical trial and those who participated and were detection of malaria infection, to be diagnosed schisotosoma positive in urine and willing to participate and follow the study procedures.**

**There were no participants with macroscopic haematuria at baseline and we did not notice any macroscopic haematuria during follow up visits.**

**Change:**

No change

--------------------

>**Results**

**Reviewer #1**: This section is clear

**Reviewer #2**: The presentation of results must provide additional information to allow a better understanding of their observed effectiveness. Indeed, there were 24 patients included in the AP group, among them 16 and 19 were followed up at week 4 and week 6. However, the

flow chart shows that 21 patients were included in the analysis. So there are 3 patients missing. In addition, the baseline data must relate to the 21 or 19 patients and not 24. The same applies to the AL group, the information provided in Table I relates to 23 patients, whereas the flow chart shows that only 18 were included in the analysis.

**Response: thank you for that comment. Data were presented as suggested.**

**Changes:**

Lines 213-214: A total of 39 out of 47 patients treated with either artesunate-pyronaridine or artemether-lumefantrine completed at least one follow up visit.

Table 1. Baseline characteristics of study participants treated with artesunate-pyronaridine and artemether-lumefantrine and included in the analysis, N=39

Line 231: Two adolescents, male and female were treated with artesunate – amodiaquine. Both had blood detected in urine with egg count of 1 and 251 eggs/10ml of urine respectively at baseline. Two school children and one adult, all of them female, received artefenomel-ferroquine as antimalarial drug with the median egg count of 124 (46 - 238) eggs/10 ml of urine at baseline.

**Reviewer**: In Table 2, the numbers at week-4 and those at week-6 differ; authors should explain why.

**Response: Some patients were lost to follow up at 4- and 6- weeks. At 4 weeks, two patients in artesunate-pyronaridine group and 6 patients in artemether-lumefantrine group were lost to follow up while two patients did not perform the visit at 6 weeks.**

**Change:**

Line 362: The overall number of participants of this observational study is limited and a proportion of patients did not completely adhere to follow up schedule.

**Reviewer**: Regarding the egg counts in Table 2, it would be more accurate to determine the parasite load in those who still excrete eggs, by excluding those who do not. This would make it possible to better estimate the reduction in egg excretion, in those who were cured and even in patients who still carry eggs (was the egg count differents between the time points, especially in those still positives?).

**Response: thank you for this relevant comment. Egg excretion reduction in patient who did not achieve parasitological cure has been performed.**

**Change:**

Line 240: Seven and 12 patients did not achieve parasitological cure at 4- and 6-weeks post-treatment respectively. Median egg excretion was 37 (IQR; 20-598) eggs/10 ml of urine with an egg excretion reduction at -10% (IQR, -633- 90) at 4-weeks, while median egg excretion was 87 (IQR; 12 - 379) eggs/10ml of urine with egg excretion reduction at -35% (IQR; -590 - 59) at 6-weeks.

**Reviewer**: The information provided in the ASAQ group and the OZFQ group do not appear to be consistent. There is only 1 positive patient in the ASAQ group, so why are there ranges presented, as well as the two % reduct’ion in excretion? Regarding the OZFQ group, the baseline eggs excretion are different but the ranges are similar.

**Response: Thank you for this comment with which we agree. ASAQ and OZFQ data has been removed in the table.**

**Change:**

ASAQ and OZFQ removed from table.

**Reviewer**: The results in Table 2 suggest an absence of impact on egg excretion in the AL and OZFQ groups at week 4. It would be interesting to have information on the presence of other symptoms and their duration. The authors should mention it in the discussion, especially since the parasite densities are generally low, apart from the AL group.

**Response: Thank you for this comment. The main and only symptom associated with schistosomiasis in our patients is haematuria. Given that patients had malaria at baseline, they had malaria related symptoms such as fever, headache, etc. but these were not reported here. Whether malaria can have some impact on the egg excretion is currently unclear and, this was mentioned in discussion section.**

**Change:**

No change

**Reviewer**: Table 3. If none of the samples from patients with low egg count were positive at week 4 post-treatment, how do the authors explain the range of 0-37 and the % shedding of -23%-100%? Were the differences observed between the groups statistically significant?

**Response: Thank you for this comment. At least half of the patients with low egg count treated with artesunate-pyronaridine achieved parasitological cure at 4-weeks and less than half of the patients achieved parasitological cure at 6-weeks as the result is presented as median. This is an observational study with no statistical hypothesis testing therefore no test for potential differences was performed.**

**Change:**

No change

--------------------

**>Conclusions**

**Reviewer #1**: Yes, this is clear

**Reviewer #2**: Although the interest of this study is undeniable, and the discussion well conducted, it addresses too succintly some important aspects. The limitations of the study should be clearly discussed :  participant selection method, the absence or presence of matching between the groups. and even the presentation of the data are major bias factors for the interpretation of the results obtained. The duration of the follow-up must be justified, because other authors report follow-up of up to several months with artemisinins and ACT. The discussion is focused on excretion rates and parasite densities. The CERMEL team has been working for several years on schistosomiasis, the authors therefore have data on the parasite loads of Schistosoma haematobium in carriers. Are they comparable to those of the participants in this study? What about the efficacy of Praziquantel after 4 or 6 weeks follow-up? There are several reports from Africa, including metaanalysis. It is important to discuss the results obtained and those of PZQ regimen, even from other studies. Doesn't the fact of not having compared the groups after the administration of praziquantel constitute another bias? Several authors have shown the superiority of the association of artemisinin derivatives with praziquantel, compared to that obtained when they are administered alone. It clearly appears that the molecules used have no action on adults, especially parous females. What do the authors think? The absence of a significant effect in high parasite load should be taken into consideration when it is known that chronic carriage which is associated with high egg count is frequent in endemic regions.

**Response: We thank the reviewer for this important comment and we agree with him/her. We consider this study as a contribution to the different proof of concepts showing the effects of artemisinin derivatives on schistosomiasis and moreover, showing also a possible effect of pyronaridine on juvenile forms as we have reported elsewhere (Kühne et al. 2021). We agree that comparison with praziquantel would be of interest but this was beyond the scope of this work. We are however preparing for a larger prospective clinical trial that may address all the relevant suggestions from the reviewer, including all the extensive work done at CERMEL on the treatment of schistosomiasis with praziquantel and other compounds.**

**Change:**

Discussion modified following the reviewer’s suggestion.

**Reviewer**: Finally, it seems that the duration of treatment with ACT alone is too short to obtain a good efficacy in reducing egg excretion. In the context of surveillance of resistance to artemisinin derivatives in Africa, what are the authors comments.

**Response: We agree with the reasoning of the reviewer that longer durations of ACT treatment would lead to higher efficacy against schistosomiasis. However, safety and tolerability of ACTs are tested only for a three day treatment, which is why we could not investigate this any further in this observational study.**

**Change:**

No changes.

--------------------

**>Editorial and Data Presentation Modifications?**

**Reviewer #1**: See attached document

**Reviewer #2:** (No Response)

--------------------

**>Summary and General Comments**

**Reviewer #1**: See attached document

**Reviewer #2**: This observationaL study is an original contribution for the integrated management of coendemic parasitic diseases with high burden in Africa. It has been ethically well conducted as consenting participantswere screened and benefitted for treatment. Finding alternatives to praziquantel, which is still the only molecule recommended for the treatment of schistosomiasis, is a priority, especially in regions of high endemicity in which this NTD and its medical, psychological and socio-economic consequences are prevalent. These are promising data in the context of the integrated approach to the management of endemic pathologies. Moreover, although the efficacy of artemisinin derivatives on schistomas has been studied for more than 10 years, such data on Schistosoma haematobium are scarce. Ethical standards are respected. The article is quite well written. However, there are too long sentences with repetitive expressions and a considerable lack of punctuation, sometimes making comprehension difficult (see lines 113 to 120, 163 to 170, 293 to 295, 306 to 310). Punctuation should be corrected in lines 112, 113 to 117, the rest of the article should be checked. A verb is missing in lines 116 to 117. The end of the sentence at line 171 needs to be reformulated.

The description of the methodology used should be improved to allo the reproductibility. Some results need to be clarified and the statistical significance is missing; If the authors can perform the revision as required, this articule should be published.

**Response: We thank the reviewer for the comments. We have checked the text and corrected the punctuation and reformulated some sentences.**

**Changes:**

For all participants a written informed consent was obtained as well acceptance for the followed up until six weeks post-treatment of malaria (post-test).

Patients were actively followed up during the 6-week period and were treated with standard PZQ therapy at the end of the follow-up period. Anti-schistosomal therapy was deferred to avoid of unknown drug-drug interaction with investigational antimalarial drugs.

--------------------

**Effectiveness of antimalarial drug combinations in treating concomitant urogenital schistosomiasis in malaria patients in Lambaréné, Gabon: A non-randomised event monitoring study**

Thank you for allowing me to review the above titled manuscript. Authors assessed the cure rates and egg reduction rates of different artemisinin combination therapies against Schistosoma haematobium. This was an observational study which was mainly a secondary analysis from the primary objective of assessing the efficacy of at antimalarial in Gabon. In general, the manuscript is well written and the methodology description are very clear. Although the manuscript does not present new knowledge on the effects of artemisinin-based antimalaria on Schistosomes species, it does add more evidence on future use of these drugs and the advantage they have in individuals who are co-infected with malaria and schistosomiasis. I have minor observations:

**Reviewer:**

- Methods: Follow the PLOS NTD Format

**Response: Thank you. Method was reported according to non randomized clinical trial (trend) guideline download from PLOS NTD page.**

**Reviewer**:

- Sample size: descriptive study, does Power make any sense?

**Response: We agree with this comment. This is a prospective observational study; therefore, no statistical hypothesis was defined. We wanted to highlight this and make it clear for readers**

**Change:**

No change

**Reviewer**:

- Urine sample collection: Did you allow patient to exercise to allow more eggs to be collected in the urinary bladder before sample collection?

**Response: Thank you for this comment. Physical exercise has been shown to increase egg output significantly, and it is therefore useful to ask patient to do some short physical exercise before collecting the urine samples. In this study patients were not advised to perform physical exercise mostly due to their ongoing acute malaria episode which precludes vigorous physical exercise. As no physical exercise was done at baseline, the same methodology was used during follow up to avoid potential bias. This has been specified in the Method and Discussion Section**

**Changes:**

Method line 162: No physical exercise prior to urine collection was advised due to the acute malaria episode.

Discussion line 366: Other limitations are the lack of robust and scalable read-outs of schistosome eggs viability after drug exposure and absence of some physical exercise before collecting the urine samples.

**Reviewer**: Another limitation is the lack of robust and scalable read-outs of schistosome eggs viability after drug exposure and the absence of some physical exercise before collecting the urine samples.

- Did you note the time of sample collection, understanding the circadian circle of eggs release?

**Response: Right, thank you for this point. Yes, time of sample collection was recorded. This information was added in the method section.**

**Change:**

Method section line 161: On the day of collection, all effort was done to collect urine between 10:00 and 15:00 as urinary excretion of the eggs follows a daily rhythm with a peak around noon. No physical exercise prior to urine collection was advised due to the acute malaria episode.

**Reviewer**:

- Assessment of ERR and CR- The WHO reference recommends 14 days to 21 days post-treatment for evaluation of ERR and CR. Why did the author assess the two variables at 4- and 6-weeks post-treatment?

**Response: Thank you for this point. It is known that an interval of 14–21 days between the treatment and the collection of follow-up data avoids the risk that eggs identified in a specimen are from parasites that infected the individual after drug administration. However, in order to standardize the follow up of patients coming from different clinical trials, repeated urine examinations were performed 4- and 6-weeks after the intake of antimalarial drug, coinciding with the patients’ visits in the antimalarial drug trial**

**Change:**

Line 159: Four and six weeks after malaria treatment coinciding with patients’ visits in the antimalarial drug trials, urine samples were collected during two consecutive days to assess participants’ eggs excretion status (post-test).

**Reviewer**:

- Results? What determined allocation of participants in different antimalaria treatment?

**Response: There are several clinical trials phase II to IV on different antimalarial drugs conducted at CERMEL during the conduct of this observational study. Each clinical trial had its own inclusion and exclusion criteria and eligible participants were enrolled in these trials. Some patients who were not consented for inclusion in clinical trials were treated routinely with first-line standard antimalarial: Arthemer-Lumefantrine or antesunate-Amodiaquine.**

**Change:**

No change

**Reviewer**:

- Did the author obtain ethical approval for this observational study?

**Response: thanks you for this point. Yes, ethical approval was obtained from the institutional ethics committee of CERMEL, reference number: CEI- 006/2018 before the initiation of the study. This was mentioned in the Ethical consideration in the manuscript.**

**Change:**

No change
